# Time optimization of gadobutrol-enhanced brain MRI for metastases and primary tumors using a dynamic contrast-enhanced imaging

**DOI:** 10.1186/s12880-022-00909-z

**Published:** 2022-10-17

**Authors:** Jeemin Seo, Changmok Lim, Kye Young Lee, Young-Cho Koh, Won-Jin Moon

**Affiliations:** 1 Department of Radiology, Konkuk University Medical Center, Konkuk University School of Medicine, 120-1, Neungdong-Ro, Gwangjin-Gu, Seoul, 05030 Republic of Korea; 2 Department of Internal Medicine, Konkuk University Medical Center, Konkuk University School of Medicine, Seoul, Republic of Korea; 3 Department of Neurosurgery, Konkuk University Medical Center, Konkuk University School of Medicine, Seoul, Republic of Korea

**Keywords:** Intracerebral metastases, Primary brain tumor, Gadobutrol, MRI, Contrast agent

## Abstract

**Background:**

Recent advances in rapid imaging techniques necessitate the reconsideration of the optimal imaging delay time for contrast-enhanced T1-weighted imaging. The aim of our study was to determine the optimal contrast-enhanced T1-weighted imaging delay time from the obtained time-signal intensity curve (TIC) using gadobutrol in patients with brain metastases, primary brain tumors, and meningiomas.

**Methods:**

This prospective study enrolled 78 patients with brain metastases (n = 39), primary brain tumors (n = 22), or meningiomas (n = 17) who underwent 7-min dynamic contrast-enhanced imaging with single-dose gadobutrol. Based on the time-to-peak (TTP) derived from the TIC, we selected four different time points for analysis. Lesion conspicuity, enhanced rate (ER) and contrast rate (CR) of 116 index lesions were evaluated. Statistical comparisons were made for the four different time points using the Friedman test.

**Results:**

Maximum TTP (305.20 ± 63.47 s) was similar across all three groups (p = 0.342). Lesion conspicuity, CR and ER increased over time in all index lesions; however, no significant difference between the 5- and 7-min images was observed. The longest diameter in all groups differed significantly among time points (p < 0.001); the perpendicular diameter did not differ between the 5- and 7-min images.

**Conclusions:**

Maximum contrast enhancement and lesion conspicuity was achieved 5–7 min after a single gadobutrol injection for brain metastases detection and for primary brain tumor/meningioma evaluation. Acquiring images 5 min after gadobutrol injection is the optimal timing for brain tumor detection during MRI work-up.

**Supplementary Information:**

The online version contains supplementary material available at 10.1186/s12880-022-00909-z.

## Background

Gadolinium-contrast enhancement magnetic resonance imaging (MRI) is an essential procedure for the diagnosis and progression determination of central nervous system (CNS) tumors [1–3]. It is still often challenging in clinical practice to distinguish single metastasis from a primary brain tumor, and occasionally meningioma [4, 5].

While various perfusion imaging techniques have being used to differentiate between brain tumor types, the contrast-enhanced T1WI (CE-T1WI) is considered the standard imaging technique [6]. Recent advances in rapid imaging techniques such as parallel imaging and compressed sensing enable us to obtain MRI sequences with a shorter acquisition time than ever [7]. Thus, the overall imaging time is shortening, forcing us to reconsider the protocol structure in a completely new way. If possible, the interval between contrast agent injection and the CE-T1WI could now be used to perform one or two short but necessary sequences. We need to know the optimal imaging waiting time for CE-T1WI, to utilize this interval better, so a contrast-enhanced fluid-attenuated inversion recovery (FLAIR) or other sequence could be acquired as a supplementary information for tumor characterization.

The conventional wisdom from a limited number of previous studies [8, 9] was that a small enhancing nodule or metastasis could be diagnosed better by delayed imaging and/or using a larger contrast agent dose. However, a retrospective observational study demonstrated that the imaging delay times at three different time points did not affect brain metastases lesion conspicuity following a single gadobutrol dose in a linear function [10]. After a single gadobutrol injection, brain metastases could be detected in both the 1- and 5-min delayed images. In contrast, the 10-min images were ineffective for detection.

A few studies using dynamic contrast-enhanced (DCE) T1-weighted perfusion imaging have vaguely suggested that the time-SI curve shape of primary brain tumors is that of enhancement with a plateau or enhancement with a later washout, not that of steady enhancement [10, 11]. However, there has been no DCE T1WI study to provide valid information regarding the optimal imaging delay time after GDCA injection. In the era of artificial intelligence-assisted radiology, it would be of importance to know the best available optimal delay time after GDCA injection in order to provide the reliable reference standard.

Therefore, we sought to characterize the time-SI curve of DCE-T1WI using gadobutrol. We also aimed to determine the optimal CE-T1WI delay time from the obtained time-SI curves in patients with brain metastases, primary brain tumors, and meningiomas. We further evaluated the DCE information qualitatively and quantitatively to determine the optimal CE-T1WI delay by extracting data at four time points (1, 3, 5, and 7 min after contrast agent injection).

## Methods

### Standards protocol approvals, registrations, and patient consents

This prospective study was approved by our institutional review board (IRB no. KUH1140098). Written informed consent was obtained from all participants prior to MR imaging. All methods were carried out in accordance with relevant guidelines and regulations.

### Patients

We recruited 120 consecutive participants with known or highly suspected brain metastases or high-grade brain tumors previously detected by computed tomography (CT) or MRI between February 2016 and June 2019. The exclusion criteria were as follows: 1) patients with a contraindication to undergo MRI; 2) patients too unstable to undergo MRI; 3) patients with a history of severe allergic or anaphylactoid reaction to contrast agents; 4) patients administered any contrast agent within 24 h before gadobutrol administration; 5) patients treated with high-dose radiation therapy any time before entering the study; 6) patients with severe cardiovascular disease; 7) pregnant or nursing female patients; 8) patients with severe renal dysfunction (glomerular filtration rate [GFR] < 30 mL/min).

**Fig. 1:**
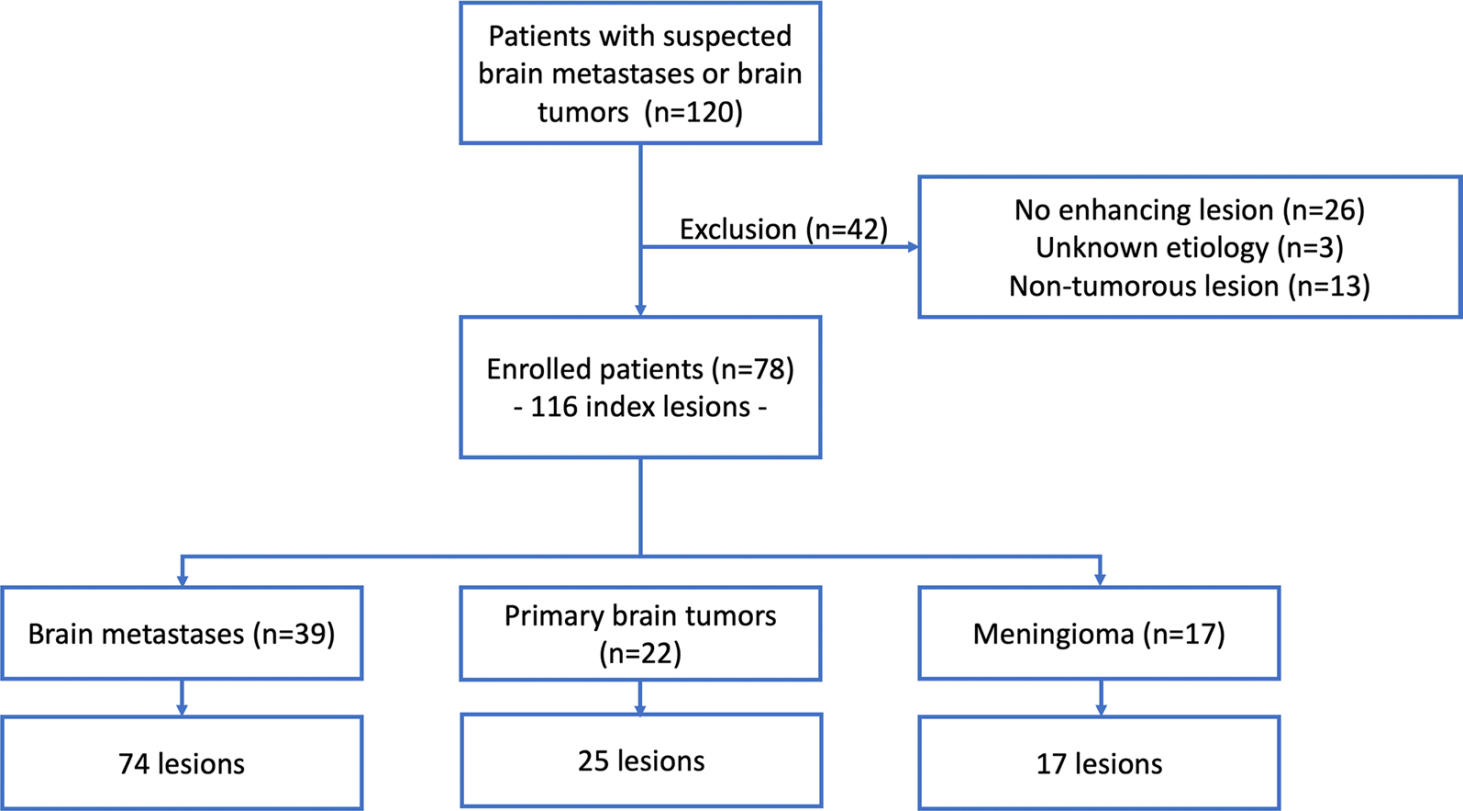
Flowchart of the study population

Of the 120 participants, we excluded those with no evidence of enhanced lesion (*n* = 26), lesion with unknown etiology (*n* = 3), and non-tumorous lesions (*n* = 13). Finally, 78 participants were considered (39 with brain metastases, 22 with primary brain tumors (grades III and IV), and 17 with meningioma). The primary origin of brain metastases was lung (n = 31, 25 with non-small cell type and 6 with small cell type), breast carcinoma (n = 6), stomach carcinoma (n = 1), renal cell carcinoma (n = 1), and urachal carcinoma (n = 1). Primary brain tumors included anaplastic astrocytoma (n = 6), glioblastoma (n = 11), central nervous system germinoma (n = 3), and primary central nervous system lymphoma (n = 2) (Fig. 1).

### MRI acquisition

Participants were scanned by the same 3 T MRI scanner (Skyra, Siemens Healthineers, Erlangen, Germany) with a 20-channel head coil. The MRI protocol included the following sequences: (1) axial 3D magnetization prepared rapid gradient echo (MPRAGE) (repetition time/echo time [TR/TE] = 1,950/3.06 ms, 0.9 × 0.9 × 0.5 mm); (2) axial 2D FLAIR (TR/TE = 9,000/95 ms, 0.8 × 0.8 × 4.0 mm); (3) axial 2D diffusion-weighted imaging (DWI; b = 1,000, TR/TE = 8,000/68 ms); (4) Axial 2D T2WI (TR/TE = 3,600/103 ms, 0.8 × 0.8 × 4.0 mm). In addition, Axial 2D FLAIR and 3D MPRAGE contrast enhanced sequences were obtained after the DCE MRI. The total acquisition time was 30 min 23 s.

### Dynamic contrast-enhanced MRI

For DCE imaging, axial 3D DCE sequence was acquired with a dynamic series of 65 individual scans with the following parameters: TR = 2.80 ms, TE = 0.90 ms, flip angle = 10°, average = 1, field-of-view = 220 mm, slice thickness = 4 mm, matrix = 160 × 148, voxel size = 0.75 × 0.75 × 4.00 mm, acquisition time = 7 min, and time resolution = 6.5 s. Pre-contrast T1-weighted gradient-echo series (TR = 2.97 ms, TE = 0.96 ms) with six different flip angles (2°–12°) were acquired to generate T1 mapping. A standard gadobutrol dose (0.1 mol/kg body weight; Bayer Healthcare), followed by a 30-mL saline flush, was automatically injected at a flow rate of 2 mL/s with an injector. Injections started after the fourth dynamic scan.

### Dynamic contrast-enhanced MRI analysis

Postprocessing and region of interest (ROI) selection in the DCE imaging data were performed by the NordicICE software (Version 4.1.3) with 3D T1-volume imaging used for structural imaging. We calculated time-to-peak (TTP) by non-model-based DCE approach. We considered the ROIs mean or maximum TTP to be the estimate of the optimal imaging delay time for lesion detection.

### Region of interest analysis for determining the lesions time-to-peak

To test our hypothesis, index lesions to be investigated in this study were first selected by CE-3D T1 MPRAGE sequence through a consensus review between two radiologists blinded to the patients’ clinical information. The size criterion for the ROI analysis was 5 mm or larger. When we found more than five lesions of the size criterion within the same patient, only five lesions were chosen for the ROI analysis. Accordingly, 74 metastatic nodules, 25 primary brain tumors, and 17 meningiomas were selected as index lesions for the ROI analysis (totaling 116 lesions). Each ROI was placed on an enhanced lesion on the CE-3D T1 MPRAGE image and then transferred to the co-registered DCE parametric map to calculate the DCE parameters mentioned above.

### Qualitative image quality assessment for the index lesion at different time points

The image slices for index lesions were collected from the DCE imaging data at four time points after contrast agent injection. The acquired images were displayed simultaneously in a random order for the qualitative assessment.

One neuroradiologist (22 years of neuroradiology experience) and one radiology trainee (3 years of radiology experience)—and both blinded to the DCE sequence image time delay and the patient clinical information—independently performed a side-by-side comparison of the four images of each index lesion to determine lesion conspicuity. The lesion conspicuity was evaluated for each image and rated on a four-point scale: 0, lesion not visualized; 1, poor visualization; 2, moderate visualization; 3, good clear visualization.

### Quantitative assessment of image quality for the index lesion according to the different time points

As an image rating quality assessment, one of the raters performed a second quantitative ROI analysis, evaluating the DCE imaging data index lesions at the four time points two weeks after the initial qualitative assessment. The ROI data was used to calculate the contrast rate (CR) and enhancement rate (ER) on images acquired 1, 3, 5, and 7 min after contrast agent injection [12]. The CR and ER of the lesion were defined as follows [12]:

$$\begin{aligned} {\text{CR}}\,\left( \% \right) & = \left( {{\text{post - contrast}}\,{\text{SI\_lesion}}{-}{\text{post - contrast}}\,{\text{SI\_white}}\,{\text{matter}}} \right) \times {1}00/{\text{post - contrast}}\,{\text{white}}\,{\text{matter}} \\ {\text{ER}}\,\left( \% \right) & = \left( {{\text{post - contrast}}\,{\text{SI\_lesion}} - {\text{baseline}}\,{\text{gray}}\,{\text{matter}}} \right) \times {1}00/{\text{baseline}}\,{\text{gray}}\,{\text{matter}} \\ \end{aligned}$$

The lesion and white matter ROIs were measured on all images, selecting the most enhanced solid portion. We strictly avoided including vessels, other strongly contrasting structures, or artifacts in the ROI. The ROI was positioned over the entire lesion enhancing area in homogeneous lesions or over the maximal enhancing area in inhomogeneous lesions. For extensive lesions with an irregular boundary, the ROI was positioned to include the largest possible proportion of the lesion at the given slice. We used the image obtained immediately after contrast injection (0 min) from the DCE data for baseline gray matter signal intensity. The ROI on the image acquired 1 min after contrast agent injection was copied to the 3, 5, and 7 min images.

Additionally, we measured the longest diameter and perpendicular diameter of the index lesions on all selected images. We chose to measure the two-dimensional diameter for the tumor size assessment because current practice such as response criteria for brain metastasis from the RANO group is still based on an unidimensional measurement [13]. Eleven lesions (seven metastases, three primary brain tumors, one meningioma) were excluded because they could not be clearly identified on images acquired 1 min after contrast injection. Thus, diameter measurements were performed on 105 index lesions.

### Statistical analysis

Statistical analysis was performed using the IBM SPSS Statistics for Windows, Version 24.0 (IBM Corp., Armonk, NY, USA) and MedCalc (Versions 19.1). Differences with *p* < 0.05 were considered statistically significant.

The Friedman test was used to evaluate the differences in index lesion conspicuity, CR, ER, diameter measurements among the four time points (1, 3, 5, and 7 min after contrast agent injection). We used the Wilcoxon rank-sum test for post hoc multiple pair-wise comparisons correction, with *p*-value < 0.008 as a Bonferroni-corrected p value.

Agreement between the two raters for lesion conspicuity was calculated using the k statistics, with k < 0.2, 0.2 to 0.4, > 0.4 to 0.6, > 0.6 to 0.8, and > 0.8 to 1.0, representing poor, fair, moderate, good, and excellent agreement, respectively.

## Result

### Determination of the peak time from the dynamic contrast-enhanced data

In brain metastases and primary brain tumors, mean TTP tended to be longer than in meningioma (185.3 ± 84.4 s and 204.32 ± 118.32 s vs. 138.61 ± 106.08 s, respectively, *p* = 0.089). However, maximum TTP was similar in all three groups (305.20 ± 63.47 s; *p* = 0.342).

### Qualitative assessment of tumor contrast enhancement at the four time points

We selected four time points to extract images from DCE data according to the TTP analysis described above. These were 1 min (60 s), 3 min (180 s), 5 min (300 s), and 7 min (420 s) after contrast agent injection.

The grade of the lesion conspicuity (degree of contrast enhancement) at the four time points after contrast agent injection showed that the lesion conspicuity increased with time in all index lesions (p < 0.001). Post hoc comparisons for all groups combined and for the metastasis group showed that the lesion conspicuity grades in the 5-min and 7-min images were similar (p = 1.000 and p = 0.234, respectively) whereas the other pair-wise comparisons were significant different (p < 0.001 for all).

Primary brain tumor and meningioma on images acquired 1 min after contrast agent injection had significantly lower lesion conspicuity grades than those on images acquired after 3 min (p = 0.008 and p < 0.001, respectively), 5 min (p < 0.001 and p < 0.001, respectively), and 7 min (p < 0.001 and p = 0.002, respectively). However, the images acquired after 3, 5, and 7 min were similar in primary brain tumor and meningioma (for 3 min vs. 5 min, p = 0.033 and p = 0.083; for 3 min vs. 7 min, p = 0.010 and p = 0.317; for 5 min vs. 7 min, p = 0.453 and p = 0.317, respectively).

Inter-rater lesion conspicuity agreement was 0.46 (95% confidence interval [CI] 0.28–0.64), 0.46 (0.27–0.64), 0.64 (0.46–0.83), 0.65 (0.48–0.81) for images acquired after 1, 3, 5, and 7 min, respectively. The inter-rater agreement of lesion conspicuity for images acquired after 5 and 7 min was better than for images acquired after 1 and 3 min.

### Quantitative assessment of the tumor contrast enhancement at the four time points

**Fig. 2:**
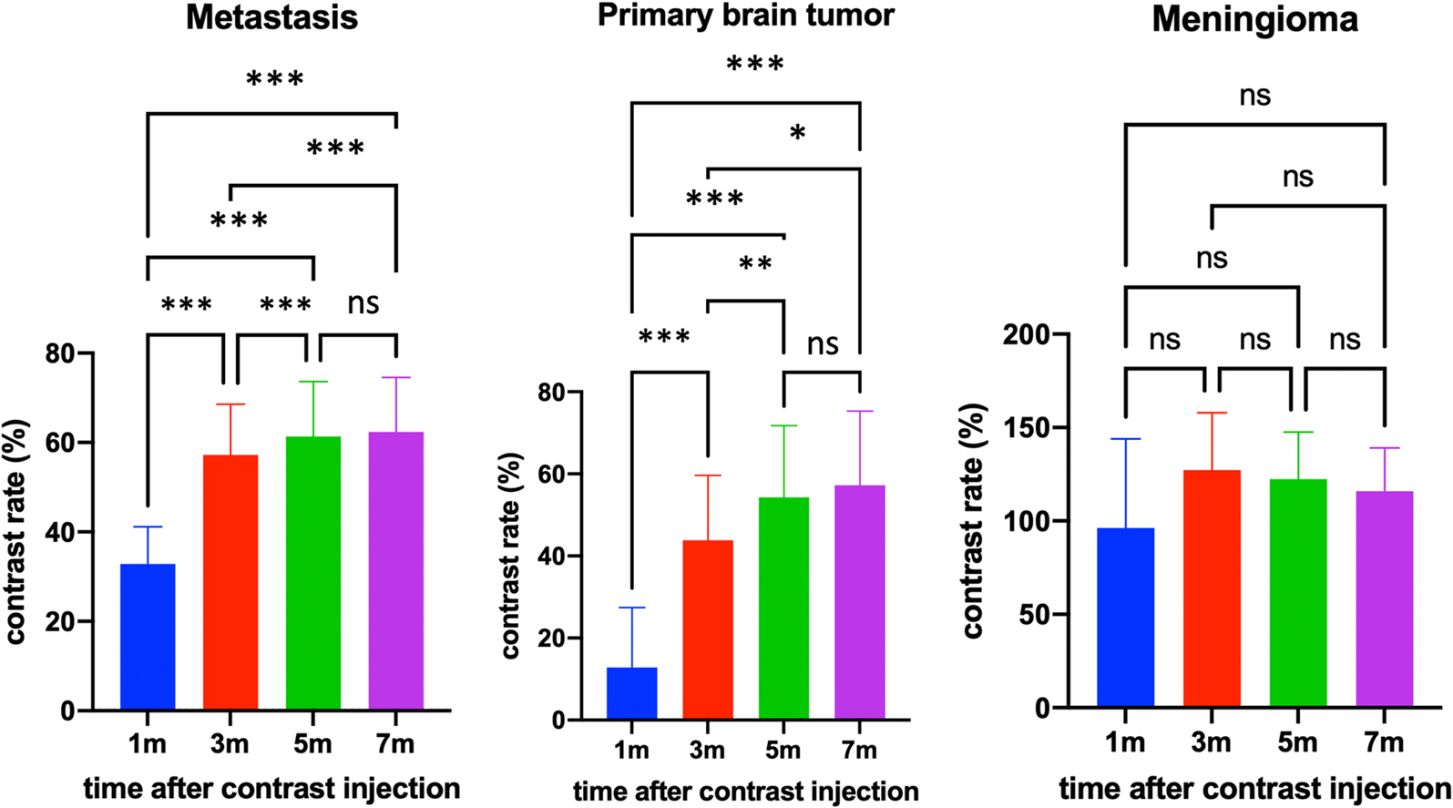
Differences in contrast rate between time points after contrast agent injection based on tumor type (*ns* not significant; * < 0.008; ** < 0.001; *** < 0.0001) statistically significant p is 0.008 as a Bonferroni-corrected p value

**Fig. 3:**
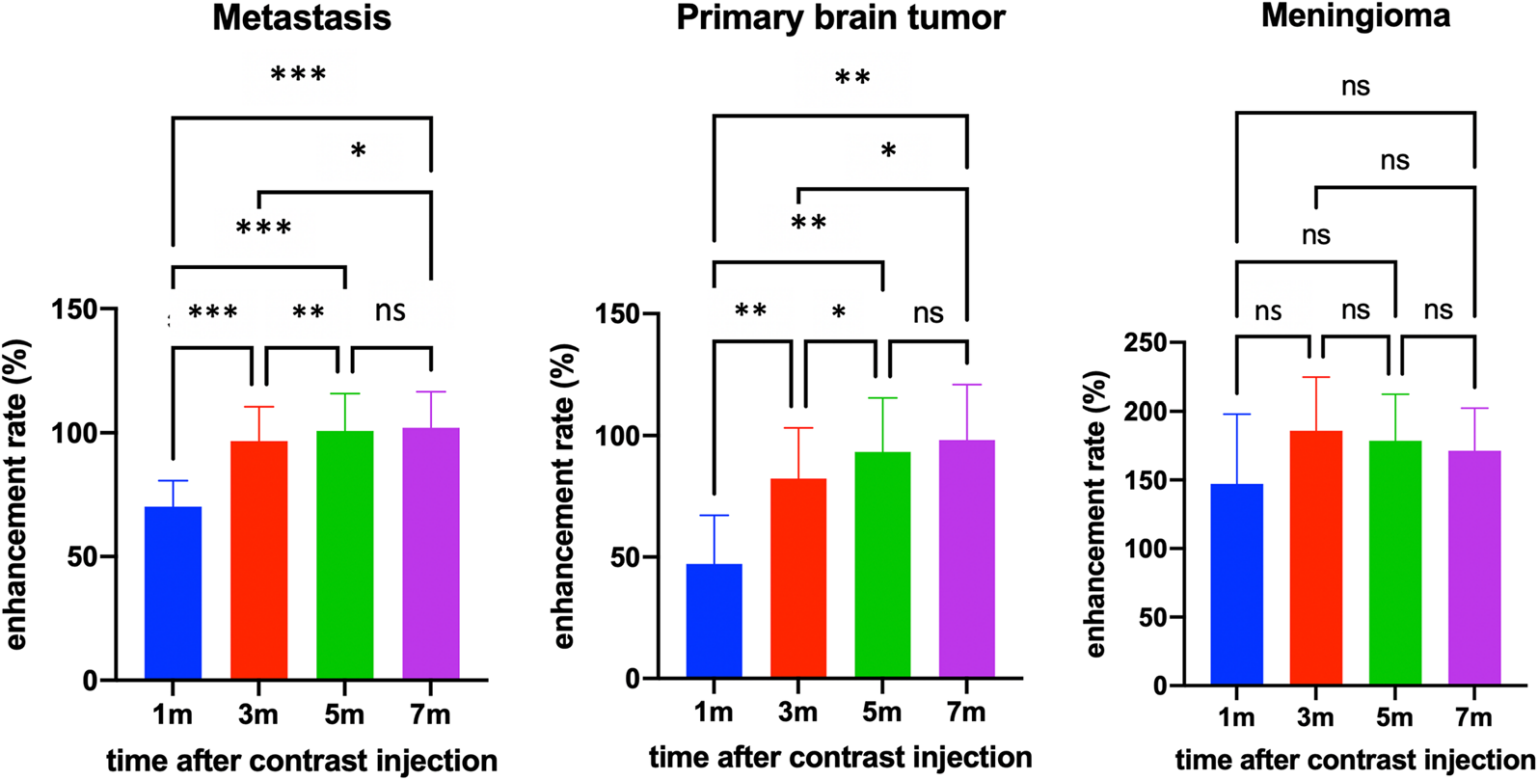
Differences in enhancement rate between time points after contrast agent injection based on tumor type (*ns* not significant; * < 0.008; ** < 0.001; *** < 0.0001)

CR and ER differed significantly between primary brain tumors and metastases images acquired 1, 3, and 5 min after contrast agent injection. However, we found no noticeable difference in CR or ER between images acquired after 5 and 7 min (Figs. 2, 3; Additional file 1: Table S1).

**Fig. 4:**
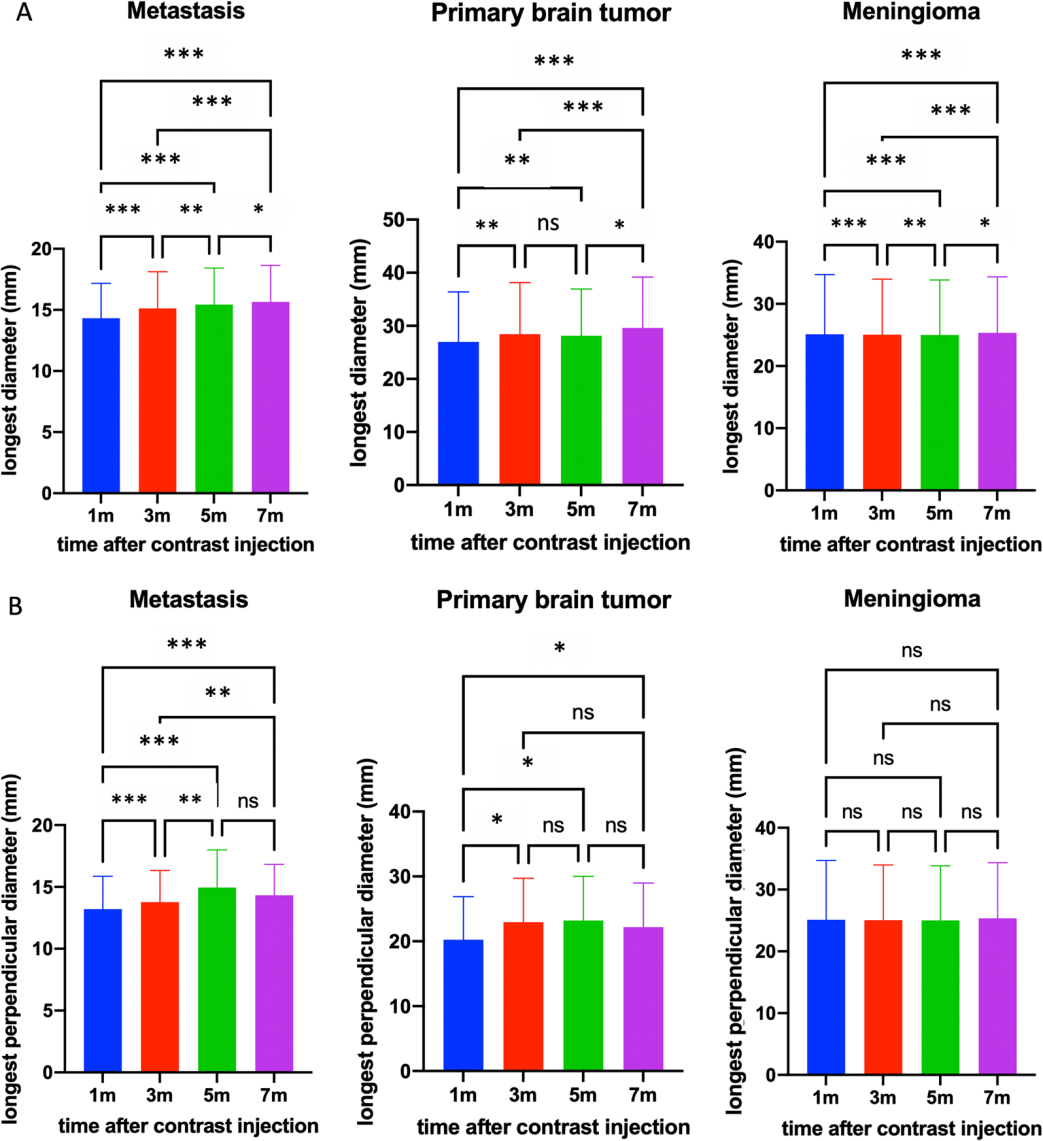
Differences between time points in the longest diameter (**a**) and the longest perpendicular diameter (**b**) (*ns* not significant; * < 0.008; ** < 0.001; *** < 0.0001)

The longest diameter in all groups differed significantly between time points (Friedman test, *p* < 0.001 for brain metastases and primary brain tumor, p < 0.019 for meningioma), except for primary brain tumors on images acquired after 3 and 5 min in pair-wise comparison (p = 0.102) (Fig. 4a). The perpendicular-longest diameter increased in size on metastases images acquired up to 5 min after contrast agent injection but was similar on images acquired after 5 and 7 min (Fig. 4b). Post-hoc comparison revealed that primary brain tumors showed no significant difference between the perpendicular-longest diameter on images acquired between 3 min, 5 min and 7 min time points. The perpendicular-longest diameter of meningioma was not different across the images acquired at all time points (Fig. 4b).

## Discussion

**Fig. 5:**
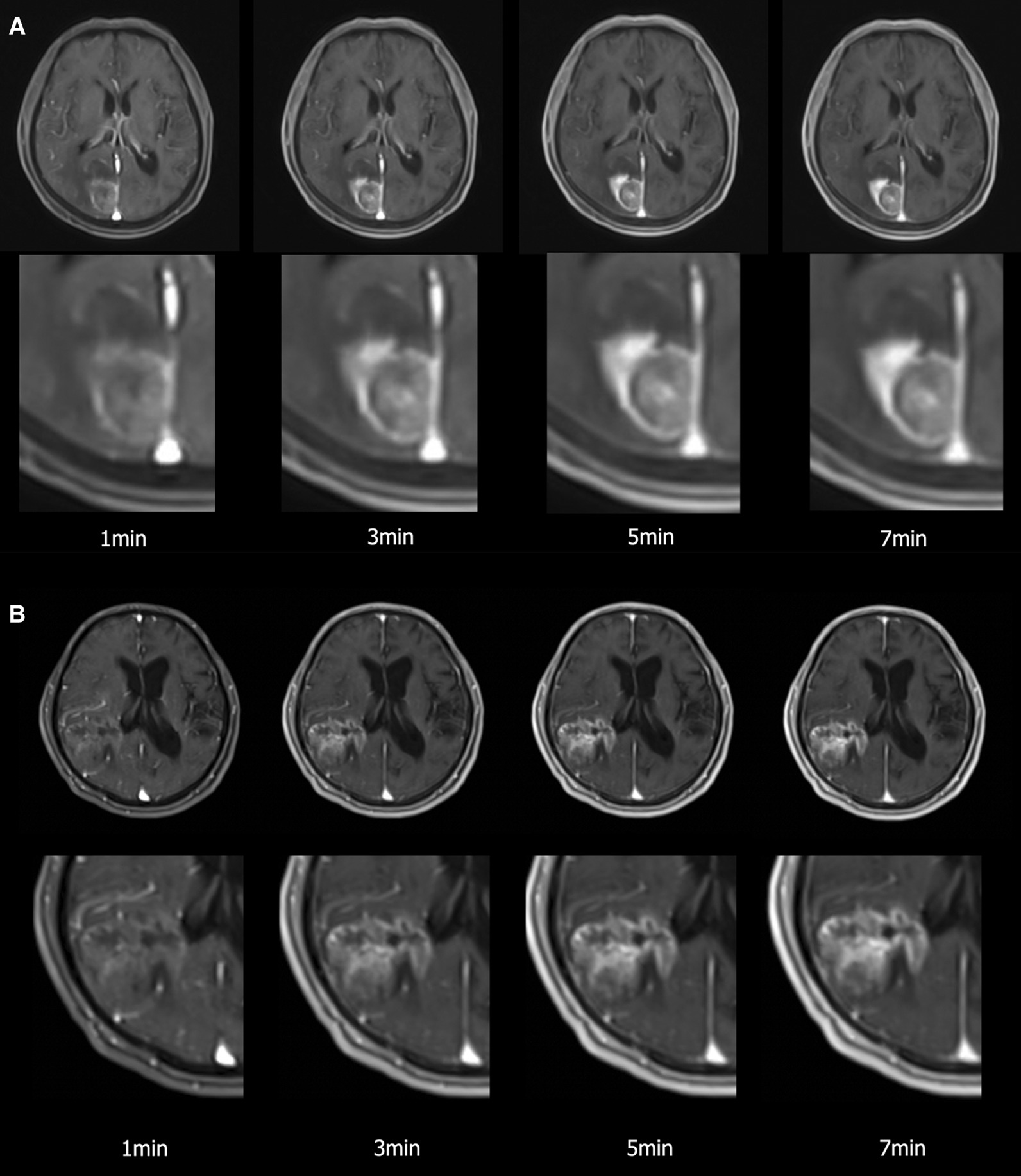
**a** A 69-year-old man displaying a brain metastasis from a lung cancer. 3D dynamic contrast enhanced (DCE) axial images acquired 1, 3, 5, and 7 min after injection of a single standard gadobutrol dose. The lesion on images acquired after 5 and 7 min appears similar in terms of conspicuity. **b** A 77-year-old man or woman displaying a primary brain tumor (glioblastoma). The lesion shows gradual increment of contrast enhancement that reaches the highest conspicuity 7 min after contrast agent injection

In this study, we found that the maximum TTP of brain metastases, primary brain tumors, and meningiomas was approximately 5 min. Qualitative and quantitative assessments of contrast enhancement supported this finding, showing that images acquired 5 and 7 min after contrast agent injection were comparable in terms of lesion conspicuity and enhancement (Fig. 5). Enhancement on images acquired after 7 min was only marginally better for the longest diameter measurement of the lesions.

Our observation of the gradual contrast enhancement in the primary brain tumor and brain metastases until 5 min after contrast agent injection when using DCE imaging partly contradicts a previous study that reported images acquired after 1 min showed an equal contrast effect to the images acquired after 5 min [10]. The researchers also found that images after 1- and 5-min delays showed a higher contrast effect than images acquired after a 10-min delay [10]. Despite our result rejecting the quality of images after 1 min, the gradual enhancement of the lesions until 5 min after contrast injection, and the comparability between images acquired after 5 and 7 min, supports the previous study’s assumption that waiting for 5 min after contrast agent injection was effective for intracerebral metastases detection [10].

Intracranial brain tumors and metastases reportedly revealed persistent or slow gradual enhancement over time [14]. However, recent DCE studies showed that primary brain tumors might exhibit early enhancement and delayed washout, not a plateau [11, 15]. Engelhorn et al. studied DCE features of experimental gliomas with 4 min temporal resolution and 30 min acquisition time and found that waiting 8 min after contrast agent injection revealed 84% of the tumors and seemed to be a practical clinical compromise between imaging delay time and detection rate [16]. In contrast, in the same study, the SNR and CNR of the tumors were highest at 4 min after contrast administration. Due to the very slow temporal resolution, the study was not able to explore the exact temporal change of contrast-enhancement between the imaging time points. A few DCE imaging studies have dealt with brain metastases [17–19], but none has determined the peak enhancement time. Our study was the first to show that the optimal brain metastases enhancement could be achieved 5 min after contrast injection based on the DCE imaging data with 6.5 temporal resolution and 7 min acquisition time. In clinical practice, the choice of contrast-enhanced MR sequence is 3D T1-weighted sequence. Whereas the acquisition times of a standard 3D T1-weighted sequences range from 4 to 5 min, those of recently developed rapid sequences are approximately 1 min [20]. For the newer rapid sequences, their narrow ranges of acquisition time may affect the final imaging quality in a negative way if the timing is not optimized.In this respect, our result can provide a valuable estimate for optimal imaging timing after contrast administration.

Differences in enhancement characteristics between the three groups might be attributable to the degree of hypervascularity, presence of dural arterial supply, difference in histologic type, and abundance of glycosaminoglycan [21]. The maximum TTP in our study was much longer than the mean TTP of the target lesion, understandable considering that the tumor enhancement curve shows a different pattern depending on the tumor portion [11]. Given tumor heterogeneity, maximum TTP might represent the entire tumor more comprehensively than mean TTP does.

The imaging assessment also confirmed that images acquired after 5 and 7 min were comparable in lesion conspicuity, ER, and CR. Our study is also in line with a previous study [10] that found lesion conspicuity and the quantitative results to become prominent over the first 5 min after contrast injection but not extending to the point of 7 min after contrast injection. However, Yuh et al. reported that small brain metastases were better detected on images acquired after a far longer waiting time (10, 20, and 30 min) [8]. Cohen-Inbar et al. also discovered more lesions 20 min after contrast agent injection, especially in association with the posterior circulation [22]. The mean diameter of the observed lesions was approximately 3 mm. Accordingly, a recent study recommended waiting 10–15 min before acquiring images to detect brain metastases [23].

However, higher sensitivity for brain metastases by any method could be at the risk of a higher false-positive rate, as when using a higher GDCA dose [24]. Besides brain metastases, solid enhancing nodules can be observed in various diseases, including vasculitis, demyelinating plaques, and infections [25]. Non-tumorous lesions with blood–brain barrier leakage can particularly benefit from delayed images, unlike brain metastases or primary brain tumors that inherently have pathological hypervascularity [26].

Our diameter measurement also revealed that waiting 5 or 7 min did not affect the measurement power of the tumor dimensions. Brain metastases response assessment is important but was not standardized until the response assessment in neuro-oncology brain metastases (RANO-BM) criteria were recently introduced [27]. Measurable disease is defined in RANO-BM as a contrast-enhancing lesion that can be measured in at least two plane dimensions, with a minimum size of 10 mm. At least a 30% decrease in the sum longest diameter of a CNS target lesion was defined as a partial response while a 20% or more increase in the sum longest diameter of a CNS target lesion was defined as progressive disease [27]. Thus, it is of paramount importance to measure accurately the tumor largest and perpendicular diameters in contrast-enhanced images. Our findings suggest that the appropriate cutoff for the waiting time from injection would be 5 min.

Our results have a potential implication in the era of AI-assisted radiology. The observations in our study could be used as a reference to develop AI-assisted diagnostic tool that deals with heterogeneously obtained imaging data. Second, our result can be used to optimize MRI protocol for quality of patient care and for throughput in MRI. In research hospitals, various sequences could be inserted between contrast agent injection and the contrast-enhanced image acquisition 5 min later. These could include dynamic susceptibility contrast (DSC), DCE, contrast-enhanced FLAIR, and more. In more rural settings, a standard enhanced FLAIR for leptomeningeal pathology could be inserted during the 5 min waiting time. Above all, accurate knowledge of the optimal waiting time could help to improve the patient’s comfort level by reducing the MRI examination time.

Our study has some limitations. First, although we evaluated the entire DCE time-intensity curve for 7 min, one could argue that estimating the time-intensity curve is inadequate. However, given the tumor pathological innate vascularity, our findings could reasonably represent the nature of the tumorous conditions studied. Second, several factors might affect the contrast enhancement, including MRI sequence parameters, post-processing software programs, and contrast agents. We acknowledge that different contrast agents, such as the macrocyclic ionic agent, might show different results. Nevertheless, given the binding nature of the macrocyclic ionic agent to glycosaminoglycan within the tumor, we assume that such a GDCA might accentuate our results rather than disapprove them [28]. The recent study revealed basically no difference in DCE time-intensity curves between gadobutrol and a macrocyclic ionic contrast agent, i.e. gadoterate meglumine [28]. Lastly, the histologic variety of primary brain tumors and the various origin of brain metastasis might affect the contrast enhancement characteristics. Further subgroup analysis was not performed because of the relatively small sample size. However, we believe that our study can be generalizable because our prospective study design allows our sample population to mirror the real-world target-population in clinical practice.

## Conclusion

In conclusion, maximum values of TTP, CR, ER, and lesion conspicuity can be achieved 5–7 min after a single-dose gadobutrol injection for brain metastases detection and for primary brain tumor/meningioma. Our findings suggest that, to optimize brain metastasis and primary brain tumor/meningioma detection on MRI workup, images should be acquired 5 min after contrast agent injection.

## Supplementary Information

**Additional file 1. Table S1:** Quantitative assessment of the index lesions.

## Data Availability

The data that support the findings of this study are available on request from the corresponding author, Moon WJ. The data are not publicly available due to their containing information that could compromise the privacy of research participants.

## Abbreviations

BBB: Blood–brain barrier
CE-T1WI: Contrast-enhanced T1WI
CR: Contrast rate
DCE: Dynamic contrast-enhanced
DSC: Dynamic susceptibility contrast
ER: Enhanced rate
FLAIR: Fluid-attenuated inversion recovery
GDCA: Gadolinium (Gd) contrast agents
MPRAGE: Magnetization prepared rapid gradient echo
ROI: Region of interest
TE: Echo time
TR: Repetition time
TTP: Time-to-peak
