# Supplementary material for: Time optimization of gadobutrol-enhanced brain MRI for metastases and primary tumors using a dynamic contrast-enhanced imaging
Source: BMC Med Imaging. 2022 Oct 17;22:180. doi: 10.1186/s12880-022-00909-z (PMC9575215; doi:10.1186/s12880-022-00909-z)
Supplement: Supplementary file 1 — Additional file 1. Table S1: Quantitative assessment of the index lesions. [file 12880_2022_909_MOESM1_ESM.docx]

Supplementary Table 1. Quantitative assessment of the index lesions.

|  | 1 MIN | 3 MIN | 5 MIN | 7 MIN |
| --- | --- | --- | --- | --- |
| The longest diameter (mm)* |  |  |  |  |
| Metastasis (*n*=67) | 14.32±11.68 | 15.99±13.04 | 16.25±13.00 | 16.40±13.07 |
| PBT(*n*=22) | 26.99±21.18 | 29.55±23.69 | 29.19±21.33 | 30.71±23.39 |
| Meningioma (*n*=16) | 25.12±17.99 | 25.65±17.78 | 25.61±17.57 | 25.96±17.90 |
| All (*n*=105) | 18.62±16.04 | 20.30±17.36 | 20.38±16.59 | 20.85±17.39 |
| The longest perpendicular diameter (mm)* |  |  |  |  |
| Metastasis (*n*=67) | 13.20±10.88 | 14.51±11.08 | 14.73±10.93 | 14.94±10.93 |
| PBT (*n*=22) | 20.23±15.00 | 23.82±16.41 | 23.98±16.50 | 22.79±16.31 |
| Meningioma (*n*=16) | 18.91±13.81 | 18.88±13.79 | 18.60±13.75 | 18.80±13.94 |
| All (*n*=105) | 15.54±12.57 | 17.13±13.19 | 17.26±13.12 | 17.18±13.04 |
| CR mean (%) |  |  |  |  |
| Metastasis (*n*=74) | 32.82 ± 35.64 | 57.21 ± 48.75 | 61.33 ± 52.74 | 62.38 ±52.09 |
| PBT (*n*=25) | 12.85 ± 34.61 | 43.81 ± 37.40 | 54.31 ± 41.36 | 57.22 ± 42.83 |
| Meningioma (*n*=17) | 96.27 ± 92.81 | 127.26 ± 59.70 | 122.42 ± 49.10 | 115.86 ± 45.31 |
| All (*n*=116) | 38.08±54.18 | 64.84±54.97 | 68.97±54.54 | 69.27±52.73 |
| ER mean (%) |  |  |  |  |
| Metastasis (*n*=74) | 74.20 ± 44.66 | 96.72 ± 59.08 | 100.75 ±64.12 | 102.02 ± 61.75 |
| PBT(*n*=25) | 47.19 ± 47.25 | 49.46 ± 49.46 | 93.35 ± 51.92 | 98.17 ± 53.52 |
| Meningioma (*n*=17) | 147.19 ± 98.87 | 186.02 ± 75.32 | 178.74 ± 65.03 | 171.40 ± 60.18 |
| All (*n*=116) | 76.84 ± 63.79 | 107.00 ± 68.28 | 110.82 ± 67.76 | 111.56 ± 64.50 |

PBT, primary brain tumor; CR, contrast rate; ER, enhancement rate.

*Eleven lesions (seven metastases, three primary brain tumors, one meningioma) were excluded because they could not be clearly identified on images acquired 1 min after contrast injection. Thus, diameter measurements were performed on 105 index lesions.

* longest diameter: pair-wise comparison not significant between 3min and 5 min

* perpendicular diameter: pair-wise comparison not significant between 5min and 7 min for metastases; not significant between 3min and 5min, between 5min and 7min, and between 3min and 7 min for primary brain tumor; not significant in all pairs for meningioma

*CR (%): pair-wise comparison not significant between 5min and 7 min for metastases and primary brain tumor; not significant in all pairs for meningioma

*ER (%): pair-wise comparison not significant between 5min and 7 min for metastases and primary brain tumor; not significant in all pairs for meningioma
